# Urine Telomerase for Diagnosis and Surveillance of Bladder Cancer

**DOI:** 10.1155/2012/693631

**Published:** 2012-07-25

**Authors:** Angela Lamarca, Jorge Barriuso

**Affiliations:** Medical Oncology Department, Hospital Universitario La Paz, 28046 Madrid, Spain

## Abstract

Bladder cancer has increased incidence during last decades. For those patients with nonmuscle involved tumors, noninvasive diagnosis test and surveillance methods must be designed to avoid current cystoscopies that nowadays are done regularly in a lot of patients. Novel urine biomarkers have been developed during last years. Telomerase is important in cancer biology, improving the division capacity of cancer cells. Even urinary telomerase could be a potentially useful urinary tumor marker; its use for diagnosis of asymptomatic and symptomatic patients or its impact during surveillance is still unknown. Moreover, there will need to be uniformity and standardization in the assays before it can become useful in clinical practice. It does not seem to exist a real difference between the most classical assays for the detection of urine telomerase (TRAP and hTERT). However, the new detection methods with modified TeloTAGGG telomerase or with gold nanoparticles must also be taken into consideration for the correct development of this diagnosis method. Maybe the target population would be the high-risk groups within screening programs. To date there is no enough evidence to use it alone and to eliminate cystoscopies from the diagnosis and surveillance of these patients. The combination with cytology or FISH is still preferred.

## 1. Introduction

Bladder cancer is a very frequent and aggressive malignant tumor. During 2011, it has been the fourth most frequent malignancy diagnosed in men and the ninth in women. Worldwide, the mortality of this tumor, three times higher in men than in women, was around 113000 deaths in men during the year 2011. The incidence increases significantly with the age, so the age-adjusted incidence rate for people under 65 years is 5,35 per 100000 habitants, and 119,76 per 100000 in people over 65 years [1].

The predominant histologic subtype found in the bladder is the transitional cell carcinoma, also known as urothelial carcinoma. The local invasion of the muscle layer in the bladder is the key prognostic factor in the approach of these patients because of the increased metastatic risk [2]. That is why the early diagnosis of the disease has a strong impact on the prognosis: those patients diagnosed earlier have a lower incidence of muscle layer affectation and use to have a better prognosis. In those patients without muscle invasion, the treatment is based on resection of the tumor by transurethral resection with adjuvant intravesical therapy (no consensus regarding the optimal drug and the optimal scheme) [3]. Second, the surgery approached for tumors with muscular involvement is radical cystectomy with bilateral pelvic lymphadenectomy. Adjuvant chemotherapy with 4 cycles of cisplatine-gemcitabine combinations is used when the tumor has reached the perivesical tissues (T3-T4) [3]. Finally, for the metastatic disease, the schedules commonly used are also gemcitabine combinations [3]. Last year, vinflunine was added to the list of drugs that have demonstrated usefulness in this setting [4–6].

## 2. Urine Biomarkers: When to Use Them?

The most common presenting symptom of patients with bladder cancer is asymptomatic microscopic hematuria or the painless macrohematuria. The percentage of symptomatic patients is difficult to say because most times the symptoms are intermittent and nondetected. Nevertheless, the early diagnosis methods based on urinary markers of bladder malignancies have been developed during the last year. There is hope to use them as early predictor of the disease, and also for the surveillance, so we could avoid the regular cystoscopy usually used for the control of the relapse of nonmuscle invasive bladder cancer [7, 8].

### 2.1. Initial Diagnosis

Urothelial cancer is usually suggested by microscopic or macroscopic hematuria and must be endoscopically excluded. However, a lot of benign lesions can produce this unspecific symptom, so even if the urine cytology could help, its low sensitivity makes a diagnostic cystoscopy required. The development of urine biomarkers could have a role in selecting those patients whom require the cystoscopy because of the higher probability of having a malignancy.

### 2.2. Surveillance

After treatment of nonmuscle invasive and superficial urothelial tumors, the high risk of recurrences makes a prolonged surveillance necessary. The gold standard test is cystoscopy and ureteroscopy. Nevertheless this semiinvasive technique that partially requires anaesthesia has got not only false negatives, but also side effects. So the design of supplementary harmless techniques as urine biomarkers could help in the surveillance of those low risk patients, in whom it could be used instead of the regular endoscopy. 

## 3. The Rationale of Using Urine Biomarkers**** and Current Status

Urine is in continuous contact with the urothelium from the renal pelvis and calyxes ureters, bladder and urethra. Thus, looking for biomarkers of malignant disease in the urine makes sense. 

The ideal diagnostic test should be noninvasive, inexpensive, easy to perform; the marker evaluated should be detected in early stage and grade tumors such as *in situ* urothelial carcinoma; the test should be highly accurate to reduce the rate of false positive and negative results.

Until now, the standard noninvasive urinary marker was urinary cytology. This technique was more sensible in high grade tumor than in low grade ones, with an overall sensitivity ranging 25–70% [28]. A lot of factors were involved in nonconsistent results, as the pathologist, the grade of the tumor, inflamative reactions of the urinary tract, test conditions, and so forth.

In the last years a great effort has been made in developing new noninvasive markers that are a hope to improve the results of the urine cytology. There are two basic methods for the study of these biomarkers: immunologic detection of soluble molecules in the urine and the analysis of the exfoliated cells from the urothelial epithelium (Table 1). With these methods we can identify proteins with increased expression in cancer cells, detect cellular antigens by immunohistochemistry or cytochemistry or identify genetic aberrations with fluorescent in situ hybridization (FISH) [7, 8]. 

Actually none of the urine biomarkers that have been studied has sufficient sensitivity to replace cystoscopy in the assessment of a suspected bladder cancer, which presents evidence to improve the results of the cystoscopy alone for the diagnosis or the surveillance of this malignancy. Additional clinical trials are necessary to determine the benefit of biomarkers and its cost-effectiveness [29, 30].

In Table 2 we summarize the most important urine biomarkers and their current stage of development [31].

## 4. Telomerase in the Pathogenesis of ****Malignancies

The role of the telomere and the telomerase in the pathogenesis of cancer has been widely studied [32–34]. 

Telomeres are repeated DNA sequences (TTAGGG) located in the 5′ ends of human cells chromosomes. This sequences loss up to 200 bases at the end of each DNA replication cycle, so they become shorter after each cellular division. The gradual loss of these repetitions plays an important role in cellular deaths, as when they disappear, the cell loses the division capacity and comes into apoptosis. Thus, telomere's shortening is related with aging and with cell's death: it is a way of controlling the number of times one cell can divide, so genetic aberrations (chromosome instability) can be avoided. The function of the telomeres is to prevent the loss of crucial genetic information. 

The telomerase is a ribonuclease-protein complex that adds bases to the telomeres to 5′ end of the chromosome, avoiding its shortening and allowing cells to replicate indefinitely. This enzyme is working in selected cells in our organism as germ cells and other continuously proliferating cells (e.g., leukocytes). Cancer cells, have reactivated this function and that is the reason they are able to live longer than noncancer cells [35]. This overfunctioning telomerase gives to cancer cells a full range of biological capabilities needed to keep dividing, growing and to disseminate. In most advanced cancers, telomerase can be reactivated and not only maintain the length of the telomere but also directly regulate cancer-promoting pathways. 

Telomerase is a reverse transcriptase that adds telomere repeats to chromosome ends. It is composed by different proteins as telomerase RNA component (TERC) and telomerase reverse transcriptase (TERT). Between them, H/ACA box is a sequence that defines a specific class of noncoding RNAs and that acts as guide for the modification of other cellular RNAs. There have been described two different types of H/ACA boxes: H/ACA small nucleolar RNAs (snoRNAs) accumulated in the nucleolus and involved in modification of ribosomal RNAs; and H/ACA small Cajal body-specific RNA (scaRNAs), accumulated in Cajal bodies and involved in the modification of splicing RNAs. The H/ACA snoRNAs and scaRNAs direct this enzyme complex to their complementary RNAs (ribosomal RNAs and splicing RNAs, resp.). Dyskerin is another essential protein for telomeres' function, as it stabilizes all the complex formed until now and allows its function. The overexpression of any of these proteins can turn into an overfunction of telomerase [36].

**Figure 1:**
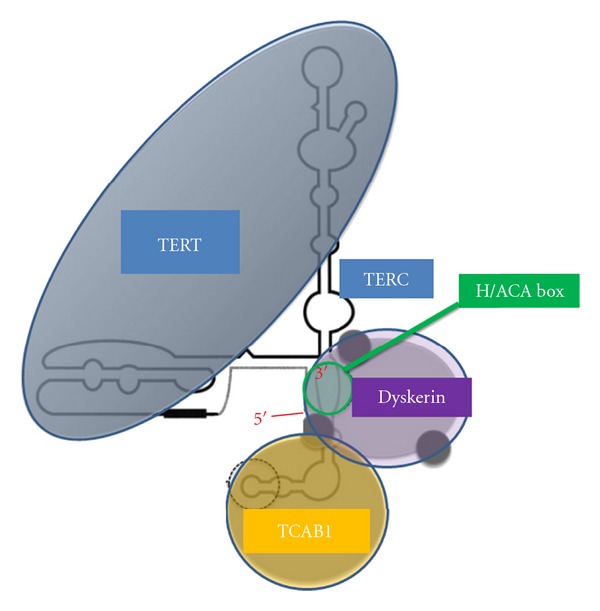
Telomerase complex.

But telomerase requires additional proteins for proper assembly and function in human cancer cells. TCAB1 is a WD40-repeat containing protein. It is associated with dyskerin and binds the CAB box in scaRNAs (not H/ACA snoRNAs or other small RNAs in Cajal bodies or nucleoli) into Cajal bodies of the cells promoting the telomerase function [37]. Depletion of TCAB1 or other protein of the complex disrupts telomerase localization to Cajal bodies and leads to progressive telomere shortening [33] (Figure 1).

## 5. Telomerase as Cancer Marker: Urine Biomarker

Telomerase is activated in 80–90% of human carcinomas [38]. The knowledge acquired by basic studies on telomere biology is being applied on the study of cancer and the development of telomerase-targeted therapies [39, 40]. 

Moreover the use of the telomerase inhibitors for cancer treatment, the telomerase quantification, has been also used, in this case, for diagnosis. Telomerase is activated in cancer cells, but not in normal somatic cells; therefore, its detection can be a diagnostic marker for cancer [38]. A recent example of this is a nonsmall lung cancer and osteosarcoma assay in which a telomerase repeat amplification protocol (known as TRAP-assay) was used. They observed that monitoring telomerase levels in blood cells could have potential applicability on diagnosis and disease surveillance [41–44].

### 5.1. Looking for the Best Diagnosis Method

After deciding to start studying telomerase as a cancer marker, multiple assays with known oncologic patients looking for the best method to detect malignancy were done. There have been designed two different ways of measuring the telomerase quantity in a tissue [43, 45, 46].

- The first one, *telomeric repeat amplification protocol (TRAP) assay*, uses PCR amplification of a telomeric template with posterior analysis by ELISA or RT-PCR. This assay needs at least 50 copies of telomerase-expressing cells to have positive results, so the false negatives are something relatively frequent [47].
- The second one, *human telomerase reverse transcriptase (hTERT) assay*, measures the messenger RNA levels of the catalytic subunit of the telomerase by RT-PCR [47].

Both assays have a sensibility between 70–100% and a specifity of 60–70% to detect cancer cells between the exfoliated cells of urine samples in symptomatic bladder cancer patients. The most used cut point is 50: so more than 50 copies of telomerase are interpreted as overexpression of telomerase [47]. Nevertheless, both can be affected by sample collection, processing, inflammation or infection with false positives results [48]. 

However, a lot of new studies are going on looking for the best diagnostic method; moreover, the combination of different urine biomarkers for urothelial cancer is being developed.

- It has been reported that human telomerase activity can be visualized by using primer-modified Au nanoparticles [49]. Even it seems to have better results; this new technique needs more investigation.
- A Japanese group designed a modified method for the detection of telomerase in urine with a simple urine telomerase activity assay by a modification of the TeloTAGGG telomerase polymerase chain reaction (PCR) enzyme-linked immunosorbent assay kit. The sensibility and specifity obtained were 81% and 92%, respectively, superior to other obtained by other groups [50].
- The combination of cytology with either urine fibronectin, TRAP, or CK20 has demonstrated higher sensibility (S 98, 4%) for diagnosis of malignancy in 132 patients with bladder cancer than cytology alone [51].
- In a series of 123 patients with bladder cancer, the combination of hTERT and cytology was superior to the combination of cytology and the measure of another urine markers by RT-PCR with a sensibility of 71% and specifity of 86% [52].

### 5.2. Looking for the Validation and Its Impact in the Clinic

The expression of telomerase in cancer cells is related with their prolonged survival. This biomarker has been studied as serum parameter in multiple diseases [41–44, 53]. We are reviewing the most important assays supporting its utility as urinary biomarker of bladder cancer. 

The usefulness of the techniques for the detection of telomerase in urooncologic patient urine has been demonstrated. As we have specified previously, they have got a sensibility between 70–100% and a specifity of 60–70% in known oncologic patients. The problem is bigger for the diagnosis of bladder cancer in patients with suspected malignancy; in this situation, few data are available yet.

The use of TRAP and hTERT seems to be similar in known oncologic urine samples. The problem is their development as diagnostic methods and the false positive index due to urinary symptoms, inflammation or infection is something to resolve. Even more, the lack of a strict relationship between hTERT protein expression and telomerase activity in validation trials has done telomerase activity in urine determined by TRAP which seemed to be a better method. Thus, TRAP has a great potential and more cost effectiveness when used, not for known oncologic patients urine malignant cellular detections, but for the diagnosis of malignancy in symptomatic patients and within high-risk subgroups [54, 55]. 

The sensibility and specificity of telomerase measure in urine for the diagnosis of urothelial/bladder cancer have been studied in a lot of different assays with different methods and results: overall the sensibility is between 60–87% and the specificity is around 65–90% [47, 56, 57]. This results increases according to stage and grade: so the positive rates are 83.3% for superficial and 42.1% for invasive stages and 83.3% for grade 1, 66.7% for grade 2, and 40.0% for grade 3 tumors; thus, telomerase activity is correlated with lower grade and lower stage bladder carcinomas [58, 59]. Specific assays done in women have confirmed this data [60]. 

Finally, as it happens in known cancer patients, the combination between different urine biomarkers improves its results. In 289 patients with urinary symptoms, the combination of TRAP and cytology, TRAP and urine FISH and TRAP with urine FISH and cytology got sensibility and specificity of 0,78 and 0,60; 0,65 and 0,93; 0,78 and 0,78, respectively, for cancer diagnosis [56]. 

### 5.3. The Actual Recommendation

Even the development of urine telomerase is quite new; the diagnostic methods have been widely studied. This is one of the advantages of using telomerase beyond other urinary biomarkers: the properly designed diagnostic tests. However, urine biomarkers have not been compared between them, so no conclusion can be drawn about which marker is the best one. The results of the assays we have summarized above of telomerase as urine biomarker of bladder cancer recommend its use in combination with other urinary biomarkers as FISH or cytology in high risk patients [61, 62]. We have not got yet enough evidence to eliminate cystoscopies from the diagnosis and surveillance of these patients.

## 6. Conclusions

Even urinary telomerase could be a potentially useful urinary tumor marker, depending on sensitivity and specifity in predetermined patients. Its use for diagnosis in symptomatic patients or its impact during surveillance is still unknown. Moreover, there will need to be normalization and standardization of the assays before they can become useful in clinical practice. Maybe the target population in whom we could use it will be in high-risk groups screening programs or as diagnosis or for surveillance programs. Nowadays there is no enough evidence to use it alone, and the combination with cytology or FISH is preferred, having said that, there is no sufficient data to avoid periodical cystoscopy.

## Figures and Tables

**Table 1:** Different methods for studying urine biomarkers.

Tests for molecular markers in urine	Tests for the analysis of exfoliated cells
Bladder tumor antigen	Automated cytology assays
Nuclear matrix protein 22	Cytokeratin 20
Nuclear matrix protein 52	Telomerase
BCLA-4 nuclear matrix protein	Microsatellite DNA
BCLA-1 nuclear matrix protein	Chromosomal abnormalities
Survivin	Carcinoembryogenic antigen
Cytokeratin 8, 18, and 19	Mucoproteins
Fibrin degradation products	Nuclear morphology abnormalities
Hyaluronic acid	DD23
Hyaluronidase	Lewis X antigen

**Table 2:** Current and emerging urinary biomarkers.

Urine biomarker	Current status of development	Reference
Bladder tumor antigen (BTA)	Bladder tumor-associated antigen in urine can be detected. The human complement factor H-related protein (hCFHrp) can be detected in the urine. Quantitative (BTA-TRAK) and qualitative (BTA-stat) assays have been done. Accepted for being used with cystoscopy. Sensitivity 50–90%; specificity 90%; false positive in urinary tract infections, calculi, benign prostatic hyperplasia and with intravesical BCG or chemotherapy (specificity 50%).	[9–11]

Genetic aberrations	(i) FISH: detection of genetic alterations in exfoliated cells in the urine (aneuploidy of chromosomes 3, 7, 17; loss of 9p21). Cystoscopy is able to detect those patients that are going to have an early relapse after all the treatment. (ii) Loss of heterozygosity and microsatellite alterations in multiple chromosomes can be detected by PCR.	[12–15]

Nuclear matrix proteins (i) NMP22 (ii) NMP52 (iii) BCLA4 (iv) BCLA1	Multiple nuclear matrix proteins are overexpressed in urothelial tumors and, after the apoptosis of these cells, released into the urine. The most studied one is NMP22, whose sensitivity is higher in high grade and in nonmuscle invasive tumors. Its levels are associated with disease recurrence and progression and it can be used together with the cystoscopy.	[16–19]

Cytokeratins (i) CK8 (ii) CK18 (iii) CK19 (iv) CK20	Cytokeratins (CKs) are proteins from the epithelial cellular cytoskeleton. Different types of CK can be over-expressed in different epithelium, so we can find specific CKs in urine in bladder cancer patients. They can be detected by ELISA or by RT-PCR. Their usefulness is under additional studies.	[15, 20, 21]

Hyaluronic acid (HA) and hyaluronidase (HAase)	Both are measured by ELISA-like assays. HA regulates cell adhesion, so it can promote tumor progression and distant metastases. HAase cuts HA into small pieces promoting angiogenesis. These markers have promising results, thus having apparent ability to detect low grade tumors better than other urine biomarkers.	[22, 23]

Survivin	An inhibitor of apoptosis that can be detected in urine by RT-PCR techniques. New studies are needed.	[24]

Others	Fibrin degradation products, DD23, Lewis X antigen.	[25–27]
